# Simultaneous Control of Venous Reservoir Level and Arterial Flow Rate in Cardiopulmonary Bypass With a Centrifugal Pump

**DOI:** 10.1109/JTEHM.2023.3290951

**Published:** 2023-06-30

**Authors:** Hidenobu Takahashi, Takuya Kinoshita, Zu Soh, Shigeyuki Okahara, Satoshi Miyamoto, Shinji Ninomiya, Toshio Tsuji

**Affiliations:** Department of Medical Science and TechnologyFaculty of Health ScienceHiroshima Kokusai Gakuin University 68402 Hiroshima 739-0321 Japan; Graduate School of Advanced Science and EngineeringHiroshima University 12803 Higashi-Hiroshima 739-8527 Japan; Department of Medical EngineeringFaculty of Health SciencesJunshin Gakuen University 74255 Fukuoka 815-8510 Japan; Clinical EngineeringHiroshima University Hospital 68272 Hiroshima 734-8551 Japan

**Keywords:** Cardiopulmonary bypass systems, automatic occluder control, two-degrees-of-freedom model matching control

## Abstract

Cardiopulmonary bypass (CPB) is an indispensable technique in cardiac surgery, providing the ability to temporarily replace cardiopulmonary function and create a bloodless surgical field. Traditionally, the operation of CPB systems has depended on the expertise and experience of skilled perfusionists. In particular, simultaneously controlling the arterial and venous occluders is difficult because the blood flow rate and reservoir level both change, and failure may put the patient’s life at risk. This study proposes an automatic control system with a two-degree-of-freedom model matching controller nested in an I-PD feedback controller to simultaneously regulate the blood flow rate and reservoir level. CPB operations were performed using glycerin and bovine blood as perfusate to simulate flow-up and flow-down phases. The results confirmed that the arterial blood flow rate followed the manually adjusted target venous blood flow rate, with an error of less than 5.32%, and the reservoir level was maintained, with an error of less than 3.44% from the target reservoir level. Then, we assessed the robustness of the control system against disturbances caused by venting/suction of blood. The resulting flow rate error was 5.95%, and the reservoir level error 2.02%. The accuracy of the proposed system is clinically satisfactory and within the allowable error range of 10% or less, meeting the standards set for perfusionists. Moreover, because of the system’s simple configuration, consisting of a camera and notebook PC, the system can easily be integrated with general CPB equipment. This practical design enables seamless adoption in clinical settings. With these advancements, the proposed system represents a significant step towards the automation of CPB.

## I. Introduction

The cardiopulmonary bypass (CPB) system is used to replace cardiac and pulmonary functions during cardiac surgery. It was first developed by Gibbon [1], and various improvements have been implemented to the devices in the system to improve safety [2], [3], [4], [5]. During surgery, perfusionists are responsible for a wide range of CPB operations, including blood flow rate control and reservoir level control. Blood flow and reservoir level regulation are closely related, and long-term training is needed to control both at the same time [6], [7].

In the CPB circuit, a patient is connected to a blood pump and reservoir via vinyl tubing attached to the arterial and venous sides, with occluders on both the arterial and venous circuits to control the blood flow rate. The amount of blood flowing into and out of the reservoir is determined by the flow rate difference between the arterial and venous sides. Because the reservoir level is correlated with the volume of blood in the patient’s heart and lungs, an unstable reservoir level can lead to unintended blood pressure fluctuations in the patient, putting the patient’s life at risk. In addition, an extreme drop in the reservoir level can lead to air embolus and cause severe damage to the patient [8], [9]. Thus, automated CPB operations that allow simultaneous control of blood flow and reservoir levels are necessary to reduce the risk of medical accidents [10].

Most studies aimed at automating blood flow rate operations have focused on CPB systems using roller pumps to control the blood flow rate [11], [12], [13]. However, there is a risk of hemolysis [14] and gaseous microemboli [15] when using roller pumps. Therefore, it is preferable to use a CPB system with a centrifugal pump. However, controlling the blood flow rate with a centrifugal pump alone [16] can lead to critical problems, such as backflow at low flow rates [17]. To address this issue, methods were proposed to automatically control the occluders so that the arterial flow rate matches the manually-adjusted venous flow rate [13], [18]. However, these systems do not control the reservoir level; thus, small disturbances can lead to fluctuations in the reservoir level, making these systems unsuitable for clinical application.

**FIGURE. 1.**
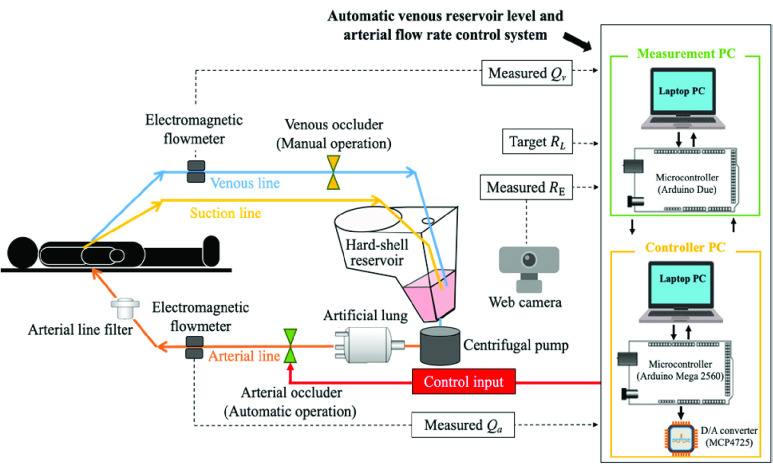
Schematic diagram of the proposed system installed in the CPB circuit. The left side shows a patient connected to a CPB circuit. The box on the right side depicts the proposed system. In the experimental setup, the connections to the patient include a venous line (blue), a suction line (yellow), and an arterial line (orange). In the experiments, a liquid tank was used to represent the patient, and a softshell reservoir was used to represent the suction field (see Fig. A3 for details). Additionally, glycerin solution or bovine blood were used as the perfusate. The orange triangles represent the manually operated venous occluder. The green triangles represent the arterial occluder, which is automatically controlled by the proposed system. The dotted lines denote the inputs to the proposed system. The flow rates measured by the flowmeters and reservoir images captured by the web camera are input to the measurement PC. The measurement PC uses the captured reservoir images to determine the reservoir level and samples the flow rates. The measured signals are input to control the PC through a microcontroller (Arduino Due). The controller PC generates and sends control signals to the arterial occluder via a microcontroller (Arduino Mega 2560). The proposed system, including the camera, laptop PC, and microcontrollers, has a straightforward design that facilitates seamless integration into general CPB systems. This integration enables the system to achieve simultaneous automatic control of both the blood flow rate and reservoir level. The simplicity of its composition further enhances its compatibility and practicality with existing CPB setups.

Therefore, in this paper, we propose an automatic system that simultaneous controls the arterial flow rate and the reservoir level, as shown in Fig. 1. The proposed system aims to control the arterial occluder to match the arterial flow rate to the given reference flow rate while maintaining the reservoir level at the desired value. A two-degree-of-freedom model matching controller [19], [20] and an I-PD controller [21] were combined and nested to achieve accurate control and suppress the influence of inevitable disturbances caused by surgery. We verified the control accuracy of the proposed system through perfusion experiments simulating the flow-up phase and flow-down phase of CPB. In this laboratory-based experiment, glycerin or bovine blood was used as the perfusate, and a liquid tank was utilized to simulate the patient. The experimental settings utilized in this study were designed based on those of previous studies on automated CPB systems, ensuring their clinical applicability [22], [23], [24], [25], [26]. The robustness of the system was then examined through experiments simulating disturbances caused by venting/suction.

## II. Materials and Methods

### A. Proposed CPB System

Fig. 1 shows a general CPB system with the proposed system installed. A CPB system is composed of a hard-shell venous reservoir, a centrifugal pump, an artificial lung, an arterial line filter, occluders to modulate blood flow rates, and electromagnetic flowmeters. The components are connected using polyvinyl chloride tubes, forming a closed circuit with the patient. The output of the centrifugal pump determines the maximum blood flow rate, and the flow rates in the arterial and venous lines are controlled using occluders.

The proposed system, as shown on the right side of Fig. 1, measures the venous flow rate and reservoir level and sends control signals to the arterial occluder. The venous flow rate is measured using an electromagnetic flowmeter, sampled by a microcontroller and input to the measurement PC. Here, the venous and arterial flow rates are denoised using a low-pass filter. The reservoir level is measured by analyzing images captured by a web camera. The image analysis procedures are described in Supplementary Material A1. The controller PC implements the proposed control system to determine the opening ratio of the arterial occluder (green triangle in Fig. 1).

### B. Controllers

**FIGURE. 2.**
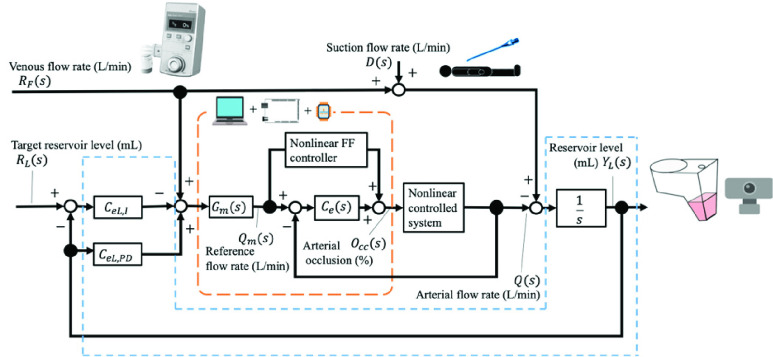
Block diagram of the proposed control system. The blocks in the orange dotted box indicate the arterial flow control unit, and the blocks in the blue box indicate the reservoir level control unit. The right upper image indicates suction from the operative field, which is a disturbance $D\left ({s }\right)$ to the system. The web camera shown on the right is used to measure the reservoir level. With these configurations, the proposed system simultaneously controls the arterial flow rate and the reservoir level based on the manually set venous flow rate (see Supplementary Material A2, Controller).

Fig. 2 shows a block diagram of the proposed system to control the opening ratio of the arterial occluder $O_{cc}(s)$, which regulates the arterial flow rate $Q\left ({s }\right)$. The input and output relationship of this control target was described using a combined linear dynamic model and nonlinear static model [27] (hereafter referred to as the nonlinear controlled system). The linear dynamic model $K_{g}$ describes the dynamic characteristics for regulating the occluder, and the nonlinear static model generates the arterial flow rate $Q\left ({t }\right)$ based on $O_{cc}\left ({t }\right)$, which is obtained by the linear dynamic model, as shown in the following equations:

\begin{align*} &\hspace {-.1pc}Q\left ({t }\right) \\ &=K_{g}\left \{{O_{cc}^{\prime }\left ({t }\right) }\right \} \\ &=\frac {{\left ({1\!+\!\frac {\Delta R_{0}}{R} }\right)Q}_{0}RA\left \{{\exp \left ({KO_{cc}^{\prime }\left ({t }\right) }\right)-1 }\right \}}{1\!+\!RA\left \{{\exp \left ({KO_{cc}^{\prime }\left ({t }\right) }\right)\!-\!1 }\right \}\!+\!\Delta R_{0}A\left \{{\exp \left ({KO_{cc}^{\prime }\left ({t }\right) \!}\right)\!-\!1 }\right \}}, \tag{1}\\ &\hspace {-.1pc}O_{cc}^{\prime }\left ({s }\right) \\ &=\frac {\exp \left ({-Ls }\right)}{1+Ts} O_{cc}\left ({s }\right), \tag{2}\end{align*}

where $O_{cc}(t)$ represents the opening ratio of the occluder, $R$ is the resistance of the entire CPB channel except the occluder channel, $\mathrm {\Delta }R_{0}$ is the resistance of the occluder channel, $Q_{0}$ is the blood flow rate with 100% occluder opening, $A$ and $K$ are parameters that express the relationship between the ratio of the crushed tube diameter to the opening ratio of the arterial occluder, $L$ is the dead time, and $T$ is the first-order time constant. These parameters were determined by a nonlinear least squares method using premeasured occluder opening ratios and arterial flow rates.

The proposed system for automatically controlling $O_{cc}(s)$ was composed of an arterial flow rate control unit and a reservoir level control unit. The arterial flow rate control unit modulated $O_{cc}(s)$ so that the arterial flow rate, $Q\left ({s }\right)$, tracked the reference flow rate, $Q_{m}(s)$. The two-degree-of-freedom model matching controller [20], [21] consists of a feedforward (FF) controller and a feedback (FB) PID controller $C_{e}\left ({s }\right)$ (see Fig. 2) and is nested in the reservoir level control unit to enable simultaneous modulation of the reservoir level and arterial flow rate. The reservoir level control unit modulated the opening ratio of the arterial occluder, $O_{cc}(s)$, so that the reservoir level, $Y_{L}\left ({s }\right)$, matched the target reservoir level, $R_{L}(s)$. The system employs an I-PD controller ${C}_{eL}(s)$ [19] composed of an I controller $C_{eL,I}\left ({s }\right)$ and a PD controller $C_{eL,PD}\left ({s }\right)$ (see Fig. 2). More details about these two controllers are presented in Supplementary Material A2.

In addition, the controllers ensured robustness against surgical disturbances induced by suction of blood from the operative field, which increases venous flow. The final value theorem can be used to prove that the effect of the disturbance on the suction flow approaches zero over time (see Supplementary Material A3 for the proof).

### C. Experiments

The first experiment (perfusion process experiment) aimed to examine the accuracy of controlling the reservoir level and arterial blood flow rate. We performed experiments simulating the perfusion process from the CPB flow-up phase to the flow-down phase for two system configurations with and without reservoir level control units. The system without the reservoir control unit was configured by removing $C_{eL}\left ({s }\right)$ and its associated inputs and outputs. In addition, two different perfusates, glycerin and bovine, were used in this experiment to test their influence on the control accuracy.

The second experiment (disturbance experiment) was performed to examine the robustness of the system against disturbances caused by venting/suction. The experimental system and experimental protocol are presented in Supplementary Material A4 and A5, respectively. The configurations used in these experiments were designed based on those applied in previous studies on automated CPB systems [22], [23], [24], [25], [26].

The efficacy of the proposed system was assessed based on two aspects, the tracking accuracy of the arterial flow rate $J_{\mathrm {Q\% }}$ and the regulation accuracy of the reservoir level $J_{\mathrm {L\% }}$, which were evaluated by the following two equations:

\begin{align*} J_{Q\%} &=\frac {1}{N}\sum \limits _{i=1}^{N} {\left |{ \frac {Q_{m}\left ({i }\right)-Q\left ({i }\right)}{Q_{m}\left ({i }\right)} }\right |\times 100}, \tag{3}\\ J_{L\%} &=\frac {1}{N}\sum \limits _{i=1}^{N} {\left |{ \frac {L_{T}-L\left ({i }\right)}{L_{T}} }\right |\times 100,} \tag{4}\end{align*}

where $J_{\mathrm {Q\% }}$ is the percentage error between the reference flow rate, $Q_{m}\left ({i }\right)$, and the measured arterial flow rate, $Q\left ({i }\right)$. Here, $i=1, 2, \cdots, N$ denotes the sampling number, where $N=s_{t} /\mathrm {\Delta }t$. $J_{\mathrm {L\% }}$ is the mean absolute error between the target reservoir level and the measured reservoir level determined using the web camera and video analysis. For all simulation experiments, $L_{\mathrm {T}}=0.45$ L, and the sampling interval was set to $\mathrm {\Delta }t$ = 0.01 s. The total measurement time was set to $s_{t}=400$ s for the perfusion process experiment and $s_{t}$ = 200 s for the disturbance experiment. The evaluation function values were compared using Fisher’s exact probability tests, with a significance level of $p < 0.05$. Statistical analyses were performed using SPSS software version 22.0.

## III. Results

**FIGURE. 3.**
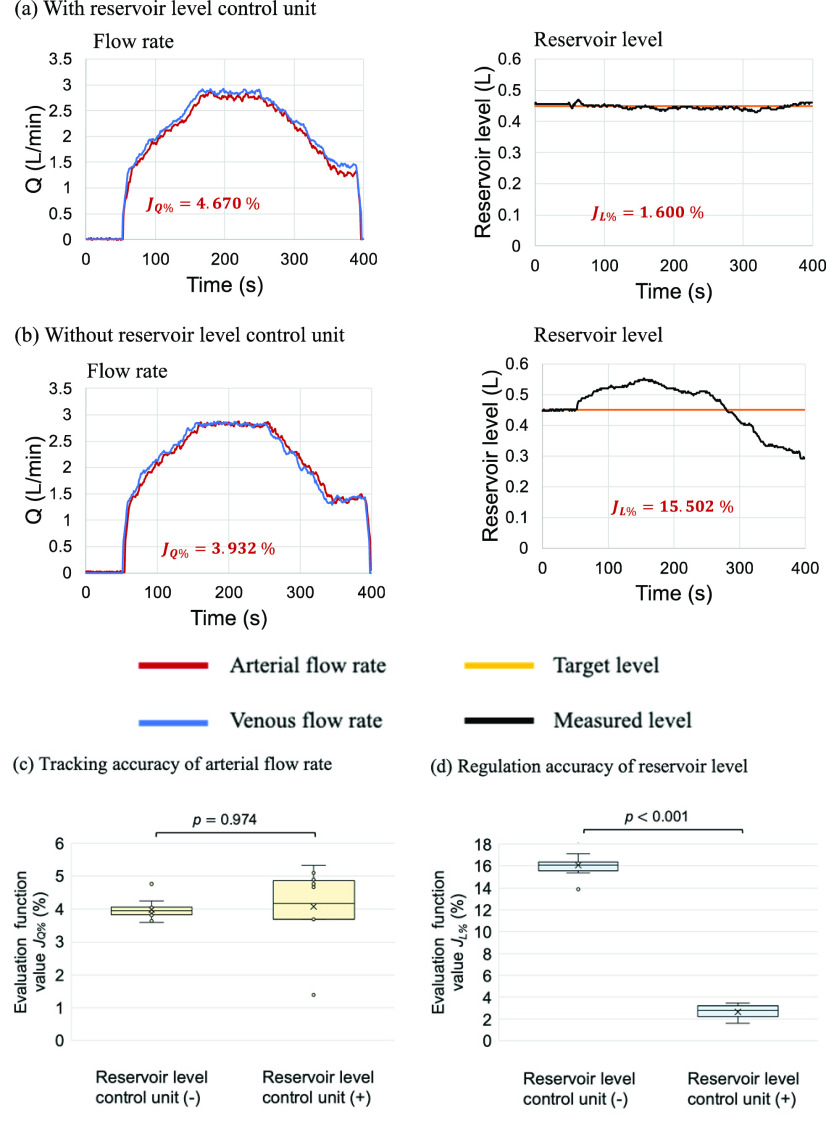
Results of the experiment simulating the perfusion process using glycerin. (a) and (b) show the control results using the proposed system with and without the reservoir level control unit, respectively. The left figure shows the flow rate. The red and blue lines in the flow rate graph indicate the arterial and reference flow rates. The right figure shows the reservoir level control results. The orange and black lines in the reservoir level graph indicate the target and measured levels, respectively. (c) and (d) show the evaluation function values obtained with the systems with and without the reservoir level control unit. Each boxplot contains the evaluation function values of 10 trials and is presented with 2.5%, 50% (median), and 97.5% quantiles and whiskers representing the minimum and maximum values. In the case with glycerin solution, the proposed control system with the reservoir control unit achieved lower errors in the reservoir level and blood flow rate.

Figs. 3 (a) and (b) show example results of the experiment simulating the perfusion process. In the two configurations of the proposed system, the arterial flow rate followed the venous flow rate, and the difference was not significant, with a percentage error $J_{\mathrm {Q\% }} < 5.32$% relative to the reference flow rate. Figs. 3 (c) and (d) present comparisons of the proposed system with and without the reservoir level control unit, showing no significant difference in $J_{\mathrm {Q\% }}$ ( $p$ = 0.97). In contrast, the system without the reservoir level control unit showed significantly worse ( $p < 0.001$) reservoir level control accuracy, with a value of $J_{\mathrm {L\% }}$ = 16.06%, while the system with the reservoir level control unit obtained a reservoir level control accuracy of $J_{\mathrm {L\% }}=2.77$%. Thus, this result indicates the importance of the reservoir level control unit.

**FIGURE. 4.**
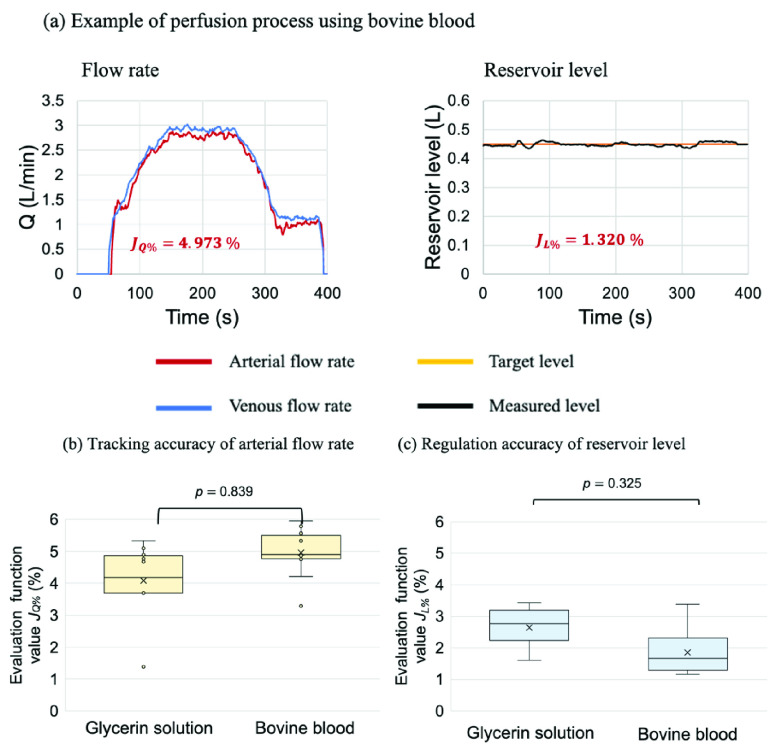
Results of the experiment simulating the perfusion process using bovine blood. The left panel of (a) shows the flow rate control results. The red and blue lines in the flow rate graph indicate the arterial and reference flow rates. The right figure in (a) shows the reservoir level control results. The orange and black lines in the reservoir level graph indicate the target and measured levels, respectively. (b) shows $J_{\mathrm {Q\% }}$, which indicates the accuracy of tracking the arterial flow rate relative to the venous flow rate. (c) shows $J_{\mathrm {L\% }}$, which indicates the accuracy of regulating the reservoir level. The blood flow control unit and the reservoir level control unit work together to provide highly accurate control in the experiments with bovine blood and glycerin solution as the perfusate.

Fig. 4 (a) shows the example results of the experiment using bovine blood with the reservoir level control unit. These examples show that the arterial blood flow rate tracked the venous blood flow rate and that the reservoir level remained nearly constant, similar to when glycerin solution was used as the perfusate. Figs. 4 (b) and (c) show a comparison of the evaluation function values obtained from the experiments with the reservoir level control unit using bovine blood or glycerin solution as the perfusate compared. These results indicate that the proposed system could simultaneously control the blood flow rate and the target reservoir level. Moreover, changing the perfusate to bovine blood did not significantly affect the control accuracy.

**FIGURE. 5.**
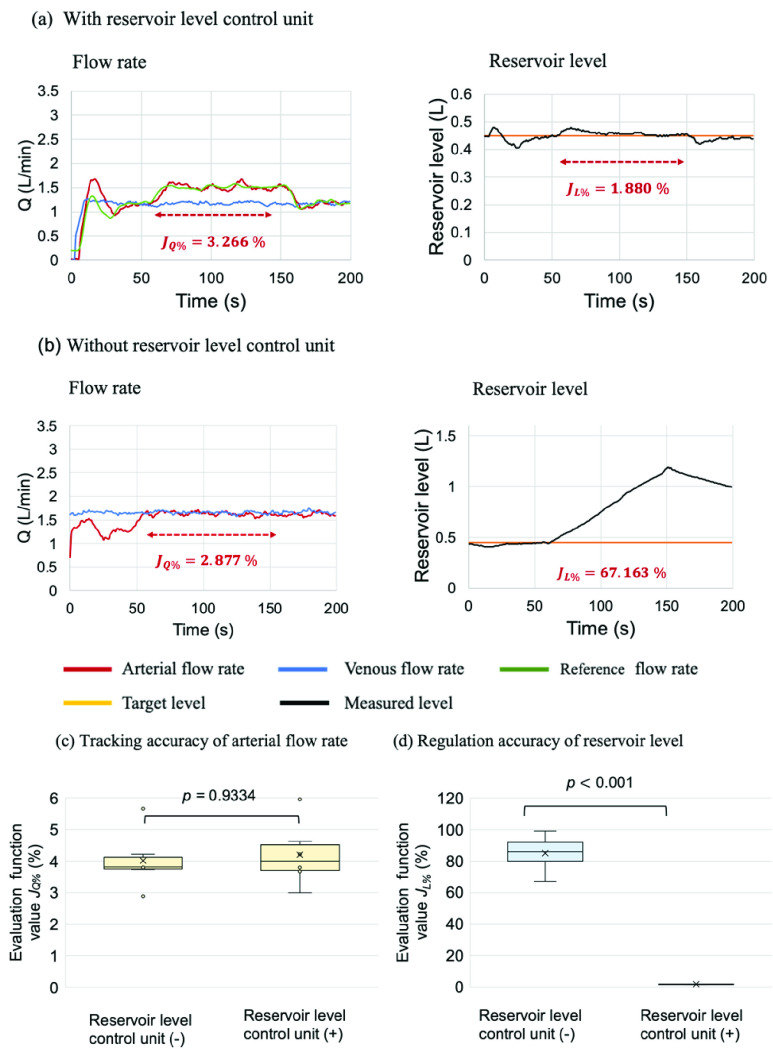
Results of the experiment simulating disturbances. (a) and (b) show the results of the proposed system with and without the reservoir level control unit, respectively. The left figure shows the flow rates. The red, blue, and green lines indicate the arterial blood flow, venous blood flow, and reference flow, respectively. The right figure shows the reservoir level. The orange and black lines indicate the target and measured reservoir levels, respectively. The red dotted arrows indicate the duration of active venting/suction. The disturbance was applied for approximately 50 and 150 seconds. (c) shows the accuracy of tracking the arterial blood flow, $J_{Q\% }$, and the right figure (d) shows the accuracy of controlling the reservoir level, $J_{L\% }$. The yellow and blue boxplots show the evaluation function values obtained by the proposed system with the reservoir level control unit (+) and without the reservoir level control unit (−), respectively. Each boxplot contains the evaluation function values of 5 trials and is presented with 2.5%, 50% (median), and 97.5% quantiles and whiskers representing the minimum and maximum values. The proposed system with the blood flow control unit and the reservoir level control unit was robust to expected disturbances.

Figs. 5 (a) and (b) show the example results of the simulated venting/suction disturbance. With venting/suction disturbance, the arterial flow rate tracked the venous flow rate with a percentage error of $J_{Q\% }=\mathrm {2.8}7$% without the reservoir level control unit, but the reservoir level deviated significantly from the target, with a percentage error of $J_{L\% }=\mathrm {67.1}6$%. In contrast, when the proposed system included the reservoir level control unit, the percentage error of the arterial flow rate was $J_{Q\% }=\mathrm {3.2}7$%, and the reservoir level was maintained at the target level with a percentage error of only $J_{L\% }=\mathrm {1.88}$%. Figs. 5 (c) and (d) show the evaluation function values of the proposed system with and without the reservoir level control unit. Fig. 5(c) shows that the system with the reservoir level control unit controlled the arterial flow rate at $J_{Q\% }=\mathrm {3.99}$% and the reservoir level at $J_{L\% }=\mathrm {1.83}$%. In contrast, the system without the reservoir level control unit had an error of $J_{L\% }=\mathrm {86.0}6$%. This result indicates that the venting/suction disturbance could cause large fluctuations in the reservoir level when the reservoir level is not controlled. Comparing the systems with and without the reservoir level control unit, $J_{Q\% }$ was not significantly different ( $p =\mathrm {0.93}$), but $J_{L\% }$ was drastically decreased ( $p < 0.001$), indicating that the proposed system could maintain the reservoir level while controlling the arterial flow rate.

## IV. Discussion

The aim of this study was to automate the control of the arterial occluder in the CPB circuit to reduce the operational burden of perfusionists, and we proposed an automatic reservoir level and arterial flow rate control system with a nested structure. Experiments were conducted to examine the control accuracy and robustness of the proposed system by simulating the perfusion process from the CPB flow-up phase to the flow-down phase and simulating venting/suction disturbances. Through these experiments, we assessed the control accuracy and robustness of the proposed system under realistic conditions encountered during clinical perfusion.

The experiment simulating the perfusion process demonstrated control accuracies of $J_{\mathrm {Q\% }} < 5.32$% and $J_{\mathrm {L\% }} < 3.44$% for glycerin perfusate and $J_{\mathrm {Q\% }} < 5.96$% and $J_{\mathrm {L\% }} < 3.39$% for bovine blood perfusate. A qualified perfusionist must control the arterial flow rate and reservoir level within a percentage error of 10% [28], and the proposed system met these requirements in terms of the control accuracy. In addition, a comparison of the accuracies obtained with different perfusates indicated that the perfusate did not significantly affect the control accuracy ( $p$ = 0.33). This suggests the potential clinical applicability of the proposed system.

In the experiment with venting/suction disturbances, the control accuracies were $J_{Q\% }=5.95$% and $J_{L\% }=2.02$%, which are less than the errors of the system operated by a skilled perfusionist. Several studies have proposed systems for controlling the arterial flow rate by tracking the manually-regulated venous flow rate [13], [16]; however, these systems ignored reservoir level control. As indicated by the venting/suction experiment, the reservoir level increases rapidly due to venting/suction disturbances, and conventional systems cannot address this problem. Changes in the reservoir level directly affect the patient’s blood pressure and can be life-threatening due to micro emboli caused by air induction. This fact highlights the importance of the reservoir level control unit. Furthermore, the robustness of the system against venting/suction disturbances can be theoretically proven by the final value theorem, with the effect of the step disturbance input approaching zero over time. This theoretical analysis also supports the robustness of the proposed system against suction/venting disturbances.

In the perfusion process using a centrifugal pump, backflow occurs at low flow rates [17], so low flow rates are controlled by an occluder, while high flow rates are controlled by the centrifugal pump. In this study, the perfusion process could be controlled by controlling the occluder with an increased centrifugal pump speed to achieve the optimal perfusion results in adults [29].

## V. Limitations

First, while we designed the experimental conditions based on those used in previous studies [22], [23], [24], [25], [26], it is possible that the configuration may not precisely replicate the conditions encountered in actual clinical practice. Therefore, additional clinical experiments involving human or animal subjects are needed to validate the findings in a real-world setting. Second, it should be noted that the CPB system in this study was not fully automated. An automatic venous flow control system is necessary to achieve a fully automated CPB system. This aspect should be considered in future studies to further advance the field and enhance the practicality of the proposed system.

## VI. Conclusion

This paper proposed a system to automatically control the arterial flow rate and reservoir level in CPB systems, with accuracies comparable to those of qualified perfusionists. Therefore, the proposed system has a high potential for clinical application to reduce errors during operation and reduce burdens on perfusionists, who are required to work long hours [30]; this may prevent accidents such as air embolization downstream of the reservoir [31], demonstrating the potential of fully automating CPB systems.

## Supplementary Materials

Supplementary materials

## References

[1] J. H. Gibbon, “Application of a mechanical heart and lung apparatus to cardiac surgery,” Minnesota Med., vol. 37, no. 3, pp. 171–185, Mar. 1954. 13154149

[2] A. Kantrowitz, S. Reiner, and D. Abelson, “An automatically controlled, inexpensive pump-oxygenator,” J. Thoracic Cardiovascular Surg., vol. 38, no. 5, pp. 586–593, Nov. 1959, doi: 10.1016/s0022-5223(19)32421-3. 14404337

[3] F. J. Lewis, S. J. Horwitz, and J. B. Naines, “Semiautomatic control for an extracorporeal blood pump,” J. Thoracic Cardiovascular Surg., vol. 43, no. 3, pp. 392–396, Mar. 1962, doi: 10.1016/s0022-5223(20)31617-2. 14464858

[4] N. Chauveau, W. Van Meurs, R. Barthelemy, and J. P. Morucci, “Automatic modules for extracorporeal circulation control,” Int. J. Artif. Organs, vol. 13, no. 10, pp. 692–696, Oct. 1990, doi: 10.1177/039139889001301011. 2254047

[5] J. Anbe, T. Tobi, H. Nakajima, T. Akasaka, and K. Okinaga, “Microcomputer-based automatic regulation of extracorporeal circulation: A trial for the application of fuzzy inference,” Artif. Organs, vol. 16, no. 5, pp. 532–538, Oct. 1992, doi: 10.1111/j.1525-1594.1992.tb00338.x. 10078307

[6] S. Ninomiya, A. Tokumine, T. Yasuda, and Y. Tomizawa, “Development of an educational simulator system, ECCSIM-lite, for the acquisition of basic perfusion techniques and evaluation,” J. Artif. Organs, vol. 10, no. 4, pp. 201–205, Dec. 2007, doi: 10.1007/s10047-007-0396-x. 18071848

[7] F. Merkle, D. Kurtovic, C. Starck, C. Pawelke, S. Gierig, and V. Falk, “Evaluation of attention, perception, and stress levels of clinical cardiovascular perfusionists during cardiac operations: A pilot study,” Perfusion, vol. 34, no. 7, pp. 544–551, Mar. 2019, doi: 10.1177/0267659119828563. 30868941

[8] J. R. Utley, “Techniques for avoiding neurologic injury during adult cardiac surgery,” J. Cardiothoracic Vascular Anesthesia, vol. 10, no. 1, pp. 38–44, Jan. 1996, doi: 10.1016/s1053-0770(96)80177-7. 8634386

[9] N. L. Mills and J. L. Ochsner, “Massive air embolism during cardiopulmonary bypass,” J. Thoracic Cardiovascular Surg., vol. 80, no. 5, pp. 708–717, Nov. 1980, doi: 10.1016/s0022-5223(19)37716-5. 7431967

[10] J. M. Toomasian, “Is it time to automate the heart lung machine?” Perfusion, vol. 36, no. 6, pp. 545–546, Aug. 2021, doi: 10.1177/02676591211039367. 34392721

[11] T. Beppu, Y. Imai, and Y. Fukui, “A computerized control system for cardiopulmonary bypass,” J. Thoracic Cardiovascular Surg., vol. 109, no. 3, pp. 428–438, Mar. 1995, doi: 10.1016/s0022-5223(95)70273-3. 7877303

[12] N. Momose, “Development of a new control device for stabilizing blood level in reservoir during extracorporeal circulation,” Perfusion, vol. 25, no. 2, pp. 77–82, Mar. 2010, doi: 10.1177/0267659110368306. 20427412

[13] Y. Niimi, S. Murata, Y. Mitou, and Y. Ohno, “Use of a novel drainage flow servo-controlled CPB for mitral valve replacement in a Jehovah’s witness,” Perfusion, vol. 33, no. 6, pp. 490–492, Mar. 2018, doi: 10.1177/0267659118763036. 29498590

[14] S. D. Hansbro, “Haemolysis during cardiopulmonary bypass: An in vivo comparison of standard roller pumps, nonocclusive roller pumps and centrifugal pumps,” Perfusion, vol. 14, no. 1, pp. 3–10, Jan. 1999. 10074641 10.1177/026765919901400102

[15] S. Yee, “Evaluation of HL-20 roller pump and rotaflow centrifugal pump on perfusion quality and gaseous microemboli delivery,” Artif. Organs, vol. 34, no. 11, pp. 937–943, Oct. 2010, doi: 10.1111/j.1525-1594.2010.01079.x. 20946282

[16] H. Nishida, “Development of an autoflow cruise control system for a centrifugal pump,” Artif. Organs, vol. 19, no. 7, pp. 713–718, Jul. 1995, doi: 10.1111/j.1525-1594.1995.tb02410.x. 8572981

[17] C. Rungsirigulnan, R. Laohasurayodhin, T. Tuanthammaruk, Y. Chusri, P. Diloksumpan, and P. Naiyanetr, “Analysis of backflow within an external centrifugal blood pump for ventricular assist device,” in Proc. 6th Biomed. Eng. Int. Conf., Oct. 2013, pp. 1–4, doi: 10.1109/BMEiCon.2013.6687729.

[18] H. Takahashi, T. Kinoshita, Z. Soh, and T. Tsuji, “Automatic control of blood flow rate on the arterial-line side during cardiopulmonary bypass,” in Proc. 43rd Annu. Int. Conf. IEEE Eng. Med. Biol. Soc. (EMBC), Nov. 2021, pp. 5011–5014, doi: 10.1109/embc46164.2021.9629644. 34892332

[19] M. Araki and H. Taguchi, “Two-degree-of-freedom PID controllers,” Int. J. Control, Automat., Syst., vol. 1, no. 4, pp. 401–411, 2003.

[20] W. A. Wolovich, Automatic Control System: Basic Analysis & Design. New York, NY, USA: Saunders College Publishing, 1994.

[21] M. Ogawa and T. Katayama, “A robust tuning method for I-PD controller incorporating a constraint on manipulated variable,” Trans. Soc. Instrum. Control Eng., vol. 34, no. 7, pp. 265–273, 1998, doi: 10.9746/sicetr1965.34.674.

[22] L. A. Sasso, K. Aran, Y. Guan, A. Ündar, and J. D. Zahn, “Continuous monitoring of inflammation biomarkers during simulated cardiopulmonary bypass using a microfluidic immunoassay device—A pilot study,” Artif. Organs, vol. 37, no. 1, pp. E9–E17, Jan. 2013, doi: 10.1111/aor.12021. 23305589 PMC3545401

[23] S. Wang, Y. Durandy, A. R. Kunselman, and A. Ündar, “A nonocclusive, inexpensive pediatric pulsatile roller pump for cardiopulmonary bypass, extracorporeal life support, and left/right ventricular assist systems,” Artif. Organs, vol. 37, no. 1, pp. 48–56, Jan. 2013, doi: 10.1111/aor.12026. 23305573

[24] R. Dhami, S. Wang, A. R. Kunselman, and A. Ündar, “In vitro comparison of the delivery of gaseous microemboli and hemodynamic energy for a diagonal and a roller pump during simulated infantile cardiopulmonary bypass procedures,” Artif. Organs, vol. 38, no. 1, pp. 56–63, Jan. 2014, doi: 10.1111/aor.12126. 23876021

[25] S. E. Olia, L. H. Herbertson, R. A. Malinauskas, and M. V. Kameneva, “A reusable, compliant, small volume blood reservoir for in vitro hemolysis testing,” Artif. Organs, vol. 41, no. 2, pp. 175–178, Feb. 2017, doi: 10.1111/aor.12724. 27087363 PMC5069150

[26] S. Sathianathan, “In vitro evaluation of Capiox FX05 and RX05 oxygenators in neonatal cardiopulmonary bypass circuits with varying venous reservoir and vacuum-assisted venous drainage levels,” Artif. Organs, vol. 44, no. 1, pp. 28–39, Jan. 2020, doi: 10.1111/aor.113404. 30512218

[27] H. Takahashi, Z. Soh, and T. Tsuji, “Steady-state model of pressure-flow characteristics modulated by occluders in cardiopulmonary bypass systems,” IEEE Access, vol. 8, pp. 220962–220972, 2020, doi: 10.1109/ACCESS.2020.3043470.

[28] A. Tokumine, S. Ninomiya, M. Tokaji, T. Kurosaki, and Y. Tomizawa, “Evaluation of basic perfusion techniques, ECCSIM-lite simulator,” J. Extra-Corporeal Technol., vol. 42, no. 2, pp. 139–144, Jun. 2010. PMC4680038 20648899

[29] G. S. Murphy and R. C. Groom, “Optimal perfusion during cardiopulmonary bypass: An evidence-based approach,” Anesthesia, vol. 108, no. 5, pp. 1394–1417 2009, doi: 10.1213/ane.0b013e3181875e2e. 19372313

[30] A. Trew, B. Searles, T. Smith, and E. Darling, “Fatigue and extended work hours among cardiovascular perfusionists: 2010 survey,” Perfusion, vol. 26, no. 5, pp. 361–370, May 2011, doi: 10.1177/0267659111409278. 21565975

[31] T. W. Willcox and S. J. Mitchell, “Microemboli in our bypass circuits: A contemporary audit,” J. Extra-Corporeal Technol., vol. 41, no. 4, pp. P31–P37, Dec. 2009. PMC4813533 20092085

