# Supplementary material for: Simultaneous Control of Venous Reservoir Level and Arterial Flow Rate in Cardiopulmonary Bypass With a Centrifugal Pump
Source: IEEE J Transl Eng Health Med. 2023 Jun 30;11:435–40. doi: 10.1109/JTEHM.2023.3290951 (PMC10393111; doi:10.1109/JTEHM.2023.3290951)
Supplement: Supplementary materials [file supp1-3290951.pdf]

Supplementary Materials

Simultaneous Control of Venous Reservoir Level and Arterial Flow Rate in Cardiopulmonary Bypass with a Centrifugal Pump

Hidenobu Takahashi, Takuya Kinoshita., *Member, IEEE.* Zu Soh., *Member, IEEE.* Shigeyuki Okahara, Satoshi Miyamoto, Shinji Ninomiya, and Toshio Tsuji., *Member, IEEE.*[E]

A1. Measurement of Reservoir Level

The reservoir images were captured by a web camera and used to determine the reservoir level through the following five steps, as shown in Fig. A1. These procedures were implemented using MATLAB (2020).

Step 1. Capturing the venous reservoir images
A web camera (ELP-USBFHD06H-SFV; Ailipu Technology Co., Ltd, Guangdong, China) was set in front of the venous reservoir to capture images of the reservoir at 30 Hz. The images were sent to the measurement PC for real-time tracking of the reservoir level.

Step 2. Registration
Image registration was performed because the camera position may differ slightly between measurements. First, a reference image was collected. Then, approximately eight geometrically corresponding points between the reference image and captured image were manually selected by displaying the images side by side on the computer screen and inputting the positions with a computer mouse. Finally, the coordinates of the captured image were projected onto the reference image using affine transformation.

Step 3. Extracting the region of interest (ROI)
A rectangular-shaped region of interest (ROI) in the reference image was determined before the experiments. The captured image was projected onto the reference image, and the corresponding ROI region was extracted.

Step 4. Counting the number of blood pixels
The pixels in the ROI, which represent blood, were extracted and counted. First, the blood pixels in the ROI were detected using
linear discriminant analysis according to the hue, saturation, and brightness vectors. The image was then binarized, with the blood pixels given values of 1 and all other pixels given values of 0. The erosion and dilation process was repeatedly performed on the binarized image to remove dot noise and fill the holes in the blood region. Finally, the number of pixels in the blood region was counted.

Step 5. Calculating the reservoir level
The blood pixel number was converted into the reservoir level using a predetermined lookup table. First, as shown in Fig. A1, a lookup table with levels from 0 to 2500 mL was generated based on the reservoir image before the experiments. The number of blood pixels was converted into the reservoir level using a second-order spline to interpolate the lookup table.

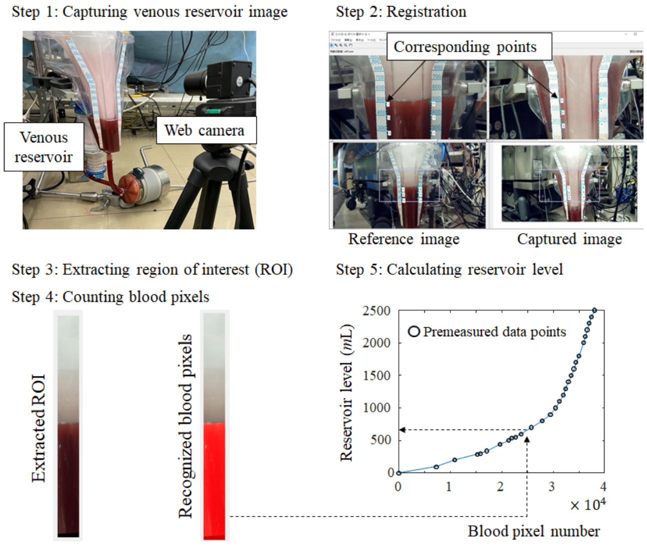

Fig. A1. Five steps to measure the reservoir level using a web camera.

Step 1 shows the experimental environment, with the web camera placed in front of the venous reservoir. Step 2 shows the PC screen for selecting the corresponding point between the reference image (left) and captured image (right). Steps 3 and 4 show the extracted rectangular-shaped region of interest and detected blood pixels, respectively. The blood pixels are highlighted in red. Step 5 shows the premeasured data points and interpolated lines representing the relationship between the blood pixel number and reservoir level. Through these sequential steps (1 to 5), the images of the venous reservoir were used to determine the corresponding reservoir level.

A2. Controller

Fig. A2 shows the equivalent block diagram of the proposed control system, consisting of an arterial flow rate control unit and a reservoir level control unit. The arterial flow rate control unit was designed based on a two-degree-of-freedom model matching control scheme, consisting of a nonlinear feedforward (FF) controller and a feedback (FB) controller. Because the control target is nonlinear (see main text Eqs. (1) and (2)), a nonlinear FF controller was constructed by combining a linear dynamic model and a nonlinear static model, which was formulated as follows:

$$O_{cc,m}(s) = \frac{(1+Ts)(1+Ls)}{\left(1+T_f s\right)^2} Q_m{}'(s) \ , \quad \text{(A1)}$$

$$\begin{aligned} Q'_m(t) &= K_g^{-1}\{Q_m(t)\} \\ &= \frac{1}{K}\ln\left\{\frac{AQ_m(t)}{\left(Q'_0 - Q_m(t)\right)R - \Delta R_0 Q_m(t)} + 1\right\}, \end{aligned} \quad \text{(A2)}$$

where $O_{cc,m}(s)$ is the reference opening ratio of the occluder and $Q_m(t)$ is the reference flow rate. Two first-order filters $\left(1+T_f s\right)^2$ were introduced to generate an appropriate transfer function. The dead time was approximated by a first-order delay $(1+Ls)$ to adapt the pole placement method in the optimization phase [1]. The reference trajectory $Q_m(s)$ input to the arterial flow rate control unit was generated assuming the following transfer function:

$$Q_m(s) = G_m(s)U_L(s) = \frac{1}{(1+\sigma s)^3} U_L(s) \ , \quad \text{(A3)}$$

where $U_L(s)$ is the target flow rate. $G_m(s)$ modeled a skilled perfusionist controlling the arterial occluder, and its behavior was specified using an arbitrary time constant σ.

The FB controller $C_e(s)$ was defined by the following transfer function:

$$C_e(s) = K_{P,e} + \frac{K_{I,e}}{s} + K_{D,e}s \ , \quad \text{(A4)}$$

where $K_{P,e}, K_{I,e}, K_{D,e}$ are the proportional, integral, and derivative gains of the arterial occluder control unit, respectively. The reservoir level control unit employs an I-PD controller $C_{eL}(s)$ composed of an I controller $C_{eL,I}(s)$ and a PD controller $C_{eL,PD}(s)$, which can be represented by the following transfer functions:

$$C_{eL,I}(s) = \frac{K_{I,L}}{s}, \quad \text{(A5)}$$

$$C_{eL,PD}(s) = K_{P,L} + K_{D,L}s \ , \quad \text{(A6)}$$

where $K_{P,L}, K_{I,L}, K_{D,L}$ are the proportional, integral, and derivative gains of the arterial occluder control unit, respectively. For a target reservoir level $R_L(s)$, the output of the I-PD controller $Y(s)$ is given by

$$\begin{aligned} Y(s) &= -C_{eL,I}(s)\{R_L(s) - Y_L(s)\} \\ &\quad + C_{eL,PD}(s)Y_L(s). \end{aligned} \quad \text{(A7)}$$

The reservoir level $Y_L(s)$ is expressed by the following equation:

$$Y_L(s) = \frac{1}{s}\left(R_F(s) + D(s) - W_F(s)U_L(s)\right), \quad \text{(A8)}$$

where $R_F(s)$ is the venous flow rate, $D(s)$ is the suction flow rate, and $W_F(s) = W_1(s)G_m(s)$. Then, the reservoir level $Y_L(s)$ can be expressed by the following equation:

$$\begin{aligned} Y_L(s) = &\frac{1 - W_F(s)}{s + W_F(s)\left(K_{P,L} + \frac{K_{I,L}}{s} + K_{D,L}s\right)} R_F(s) \\ &+ \frac{\frac{K_{I,L}}{s} W_F(s)}{s + W_F(s)\left(K_{P,L} + \frac{K_{I,L}}{s} + K_{D,L}s\right)} R_L(s) \\ &+ \frac{1}{s + W_F(s)\left(K_{P,L} + \frac{K_{I,L}}{s} + K_{D,L}s\right)} D(s) \ , \end{aligned} \quad \text{(A9)}$$

where $K_{P,L}, K_{I,L}, K_{D,L}$ are the proportional, integral, and derivative gains of the reservoir level control unit, respectively.

The parameters of the controllers are determined using the pole placement method. The closed-loop transfer function in the PID controller $W_1(s)$ (see Fig. A2) can be expressed by the following equation:

$$W_1(s) = \frac{G(s)\left(C_{fl}(s) + C_e(s)\right)}{\left(1 + G(s)C_e(s)\right)}, \quad \text{(A10)}$$

where $C_{fl}(s)$ is the linearized FF controller. Here, $G(s)$ was approximated as follows to allow for the adoption of the pole placement method.

$$\begin{aligned} G(s) = \frac{K_{gl}}{1+Ts} e^{-Ls} &\cong \frac{K_{gl}}{1+Ts}\frac{1}{1+Ls} \\ &= \frac{K_{gl}}{1 + \alpha_1 s + \alpha_2 s^2} \ , \end{aligned} \quad \text{(A11)}$$

where e^{-Ls} is the dead time parameter, Ts is the first-order time constant, $K_{gl} = dQ(t)/dO_{cc}(t)$, $\alpha_1 = T + L$, and $\alpha_2 = TL$. Substituting Eqs. (A4) and (A11) into (A10) yields $W_1(s)$

$$= \frac{\frac{K_{D,e}}{K_{I,e}}s^2 + \frac{K_{P,e}}{K_{I,e}}s + 1}{1 + \frac{1 + K_{gl}K_{P,e}}{K_{gl}K_{I,e}}s + \frac{\alpha_1 + K_{gl}K_{D,e}}{K_{gl}K_{I,e}}s^2 + \frac{\alpha_2}{K_{gl}K_{I,e}}s^3} \ . \quad \text{(A12)}$$

In addition, the reference model $G_m(s)$ can be rewritten according to the following equation using the parameter σ:

$$G_m(s) = \frac{1}{1 + 3\sigma s + 3\sigma^2 s^2 + \sigma^3 s^3} \ . \quad \text{(A13)}$$

Because the denominators in Eq. (A12) and Eq. (A13) have the same order, the PID gains in $W_1(s)$ can be determined as follows through the use of the pole placement method:

$$\begin{aligned} K_{P,e} &= \frac{1}{K_{gl}}\left(\frac{3\alpha_2}{\sigma^2} - 1\right), \\ K_{I,e} &= \frac{\alpha_2}{K_{gl}\sigma^3} \ , \\ K_{D,e} &= \frac{1}{K_{gl}}\left(\frac{3\alpha_2}{\sigma} - \alpha_1\right). \end{aligned} \quad \text{(A14)}$$

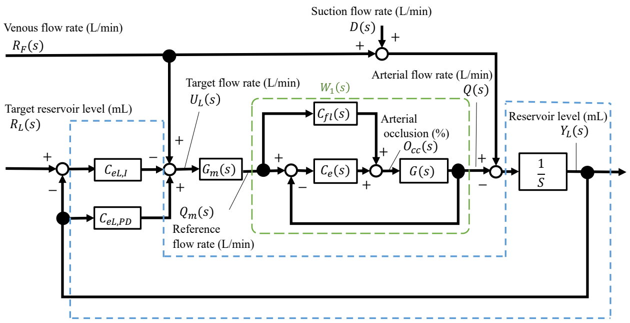

Fig. A2. The equivalent block diagram of the proposed control system.

The green dotted box indicates the arterial flow rate control unit, while the blue dotted box indicates the venous reservoir level control unit. Fluctuations in the suction flow rate $\boldsymbol{D(s)}$ and the control error of the arterial flow rate can cause variations in the reservoir level. To address this issue, the reservoir level control unit effectively manages and suppresses these fluctuations. Its primary functions are to prevent blood pressure instability in the patient and minimize the risk of air injections during the perfusion procedure.

The pole placement method was also applied to determine the gain parameters of the I-PD controller used in the reservoir level control unit. Approximating $G_m(s) \sim 1/(1+3\sigma s)$ and substituting this approximation into the transfer function of the I-PD controller, Eq. (A9), yields the following expression:

$$Y_L(s) = \frac{\frac{3\sigma}{K_{I,L}}s}{1+\frac{K_{P,L}}{K_{I,L}}s+\frac{1+K_{D,L}}{K_{I,L}}s^2+\frac{3\sigma}{K_{I,L}}s^3}R_F(s) + \frac{1+3\sigma s}{1+\frac{K_{P,L}}{K_{I,L}}s+\frac{1+K_{D,L}}{K_{I,L}}s^2+\frac{3\sigma}{K_{I,L}}s^3}R_L(s) + \frac{\frac{1}{K_{I,L}}s(1+3\sigma s)}{1+\frac{K_{P,L}}{K_{I,L}}s+\frac{1+K_{D,L}}{K_{I,L}}s^2+\frac{3\sigma}{K_{I,L}}s^3}D(s). \tag{A15}$$

Because the denominators of Eq. (A13) and Eq. (A15) have the same order, the gain parameters can be determined as follows:

$$K_{P,L} = \frac{9}{\sigma}, \quad K_{I,L} = \frac{3}{\sigma^2}, \quad K_{D,L} = 8. \tag{A16}$$

A3. Robustness against Disturbances

The influence of venting/suction disturbances was examined. Because the nonlinear FF controller and FB controller compensated for the nonlinearity and modeled dynamic properties of the nonlinear controlled system (see Fig. A2), the overall transfer function of the arterial flow rate control unit can be reduced to 1. In Eq. (A9), the third term, denoted as $Y_D(s)$ as shown below, corresponds to the influence of the venting/suction disturbance.

$$Y_D(s) = \frac{\frac{1}{K_{I,L}}s(1+3\sigma s)}{1+\frac{K_{P,L}}{K_{I,L}}s+\frac{1+K_{D,L}}{K_{I,L}}s^2+\frac{3\sigma}{K_{I,L}}s^3}D(s). \tag{A17}$$

Assuming $D(s)$ as a unit step disturbance and applying the final value theorem yields

$$\begin{aligned} y_d(\infty) &= \lim_{s\to 0} s\,Y_D(s) \\ &= \lim_{s\to 0}\left(\frac{\frac{1}{K_{I,L}}s(1+3\sigma s)}{1+\frac{K_{P,L}}{K_{I,L}}s+\frac{1+K_{D,L}}{K_{I,L}}s^2+\frac{3\sigma}{K_{I,L}}s^3}D(s)\right) \\ &= 0. \end{aligned} \tag{A18}$$

This proves that the influence of venting/suction disturbance approaches zero over time.

A4. Experimental System

Fig. A4 illustrates the experimental system composed of the CPB circuit and the proposed system. The patient's body was simulated using a soft-shell reservoir and liquid tank in all experiments, and other settings were configured as in the clinical CPB system. A centrifugal pump was used to pump blood, and an artificial lung (NHP®; Senko Medical Instrument Mfg. Co. Instrument Mfg. Co, Ltd, Tokyo, Japan) and an arterial line filter (HAF-A1; Senko Medical Instrument Mfg) were also used. The components in the CPB circuit were connected using polyvinyl chloride tubes. Electromagnetic flowmeters (FD-M5AT; Keyence Corp, Tokyo, Japan) were attached to the arterial line (3/8 inch tube) and the venous line (1/2 inch tube) to measure the flow rate at a sampling frequency of 100 Hz. A web camera (ELP-USBFHD06H-SFV; Ailipu Technology Co., Ltd, Guangdong, China) was used to capture images of the venous reservoir at 30 Hz. A third-order Butterworth low-pass filter with a cutoff frequency of 5 Hz was employed to denoise the measured flow rate data. The measured flow rates and reservoir images were then input into the proposed system, and signals were generated to control the arterial occluder (HAS-RH200; Senko Medical Instrument Mfg. Co., Ltd., Tokyo, Japan).

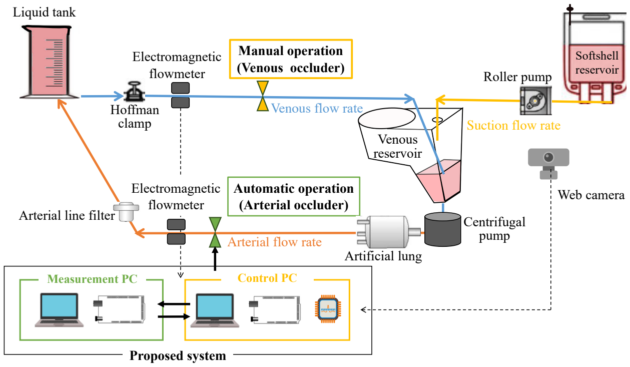

Fig. A4. Schematic diagram of the experimental system for the simulation experiments
The blue, orange, and yellow lines correspond to the venous, arterial, and suction lines, respectively. The dashed lines represent the inputs to the proposed system, which is shown at the bottom. The liquid tank shown in the upper left corner was used to represent the patient. The softshell reservoir and roller pump in the upper left corner were used to simulate suction flow. Except for these replacements, the configuration of the proposed experimental system closely resembles that used in clinical practice. This setup allows for the comprehensive simulation of the entire perfusion procedure, covering all stages from start to end.

The glycerin solution was prepared by adding glycerin and red powdered paint (Dianix Red 200%; Mitsubishi Chemical Corp, Tokyo, Japan) to water. The viscosity was measured with an oscillating viscometer (SV-10; A&D Co., Ltd., Tokyo, Japan) and was found to be 2.84 mPa·s at 18.8 degrees Celsius. The bovine blood solution was prepared by mixing bovine red blood cell solution, the anticoagulant sodium citrate (K-10 Funakoshi Co., Ltd., Tokyo, Japan), and saline. The viscosity was 2.66 mPa·s at 24.3 °C, and the hematocrit was 25.0%.

Venting/suction was simulated by using a roller pump (MF-02; JMS Co., Ltd., Hiroshima, Japan) to transfer perfusate from the softshell reservoir (upper right of Fig. A4) to the venous reservoir (HVR-4NFP; Senko Medical Instrument Mfg. Co., Ltd., Tokyo, Japan). Glycerin and bovine blood were used as perfusates.

A5. Experimental Protocol

Before the experiments, the venous reservoir was filled with 3.0 L of glycerin solution or bovine blood, which were used as perfusates. The potential for clinical application was evaluated by determining whether the characteristics of the perfusates affect the control accuracy. The maximum venous and arterial flow rates were adjusted from 1.0 to 3.0 L/min using a Hoffman clamp and a centrifugal pump with a rotational speed of 1700-2200 rpm. The target reservoir level was set to 0.45 L.

The perfusion experimental protocol was designed to simulate the flow-up, flow maintenance, and flow-down phases of CPB. For the flow-up phase, the flow rate was increased from 0.0 L/min to 3.0 L/min in 150 seconds. The flow maintenance phase was then allowed to run for 100 s. During the flow-down phase, perfusion was stopped by decreasing the flow rate from 3.0 L/min to 1.0 L/min in 100 seconds, maintaining the flow rate at 1.0 L/min for 50 seconds and finally instantly decreasing the flow rate to 0 L/min. This protocol was repeated for 8 trials for each system configuration (with and without the reservoir level control unit) and each perfusate (glycerin and bovine).

The disturbance experimental protocol was designed to simulate venting/suction. Venting/suction is a surgical procedure in which blood from a bleeding surgical field is collected and returned to the venous line to restore the extracorporeal circulating blood volume. The venting/suction disturbance was simulated by adding 0.35 L/min of perfusion flow directly to the venous reservoir for 100 s after stabilizing the CPB system. This experiment was repeated for 5 trials for each system configuration.

REFERENCES

[1] M. Kinnaert and Y. Peng, "The design of digital pole placement controllers," *Digit. Control Sys. Implementation Techn.*, vol. 70, pp. 25–65, 1995, doi: 10.1016/S0090-5267(06)80026-5.
